# KEEPING UP WITH THE JONESES: SOCIOECONOMIC STATUS AND SUBJECTIVE PERCEPTIONS OF AGING

**DOI:** 10.1093/geroni/igz038.2670

**Published:** 2019-11-08

**Authors:** Jennifer A Bellingtier, Alaina N English, Shevaun D Neupert

**Affiliations:** 1 Friedrich Schiller University Jena, Jena, Germany; 2 North Carolina State University, Raleigh, North Carolina, United States

## Abstract

Money may not buy happiness, but can it buy a more positive outlook on aging? Past research examining the associations between indicators of socioeconomic status (SES) and subjective perceptions of aging have been mixed with some finding greater socio-economic resources predict more positive aging perceptions, whereas others find no connection. We examine objective (i.e., income and education) and subjective social status (i.e., MacAurthur ladder) and their connections to subjective age, attitudes towards aging, and awareness of age-related changes using hierarchical multiple regression analyzes. Participants (n =296, age range 60-90) completed survey measures online. Results indicate minimal connection between income, education, and aging perceptions. However, perceiving oneself to be higher in social standing compared to one’s community was consistently related to more favorable perceptions of aging. Higher community standing may indicate favorable development, fewer stressors, or more resources compared to others, which could contribute to more positive perceptions of aging.

